# Lectins of *Mycobacterium tuberculosis* – rarely studied proteins

**DOI:** 10.3762/bjoc.15.1

**Published:** 2019-01-02

**Authors:** Katharina Kolbe, Sri Kumar Veleti, Norbert Reiling, Thisbe K Lindhorst

**Affiliations:** 1 Tuberculosis Research Section, Laboratory of Clinical Immunology and Microbiology, National Institute of Allergy and Infectious Diseases, 33 North Drive, Bethesda, 20892, MD, United States; 2 Microbial Interface Biology, Research Center Borstel, Leibniz Lung Center, Parkallee 22, 23845 Borstel, Germany; 3 German Center for Infection Research (DZIF), Borstel Site, 23845 Borstel, Germany; 4 Otto Diels Institute of Organic Chemistry, Christiana Albertina University of Kiel, Otto-Hahn-Platz 3–4, 24118 Kiel, Germany

**Keywords:** adhesion, carbohydrates, fimbriae, lectins, *Mycobacterium tuberculosis*, pili

## Abstract

The importance of bacterial lectins for adhesion, pathogenicity, and biofilm formation is well established for many Gram-positive and Gram-negative bacteria. However, there is very little information available about lectins of the tuberculosis-causing bacterium, *Mycobacterium tuberculosis* (*Mtb*). In this paper we review previous studies on the carbohydrate-binding characteristics of mycobacteria and related *Mtb* proteins, discussing their potential relevance to *Mtb* infection and pathogenesis.

## Introduction

More than 135 years after the discovery of *Mycobacterium tuberculosis* (*Mtb*) by Robert Koch [1], tuberculosis (TB) is still one of the world’s deadliest communicable diseases [2]. TB is theoretically curable and preventable, especially since effective antibiotics have been available since the 1940s [3–5]. However, the World Health Organization (WHO) reported 1.6 million fatalities worldwide from tuberculosis in 2017, with more than 10 million annual new cases, and an overall estimated global burden of almost 1.7 billion latently infected people [2]. The fight against this primarily pulmonary disease is strongly influenced by localized poverty and the efficiency of regional health care systems, and nowadays is further complicated by the rapidly increasing prevalence of antibiotic-resistant *Mtb* strains [2]. To successfully combat this disease, it is important to improve our understanding of *Mtb* biology and identify new drug targets and anti-*Mtb* strategies.

*Mtb* bacteria are mainly transmitted by inhalation of aerosolized droplets released from infected patients by coughing. The infection process is initiated by contact between inhaled bacteria and host cells within the alveolar airspace. The main target cells of *Mtb* bacteria are primarily alveolar macrophages, which internalize the pathogen through phagocytosis [6]. These innate immune cells initiate a number of responses to limit bacterial replication and spread with the ultimate goal of eradicating the pathogen. However, *Mtb* has evolved successful strategies to survive, replicate and persist within macrophages for days, months or even years, including highly-specialized metabolic pathways for nutrient acquisition and stress-responsive processes for protection against the immune system [7–12]. In this regard, invasion of alveolar macrophages is considered as one of the seminal steps in *Mtb* infection. However, within the alveolar space of the lung, epithelial cells are present in far larger numbers than macrophages. The first cells that *Mtb* encounters are therefore most likely alveolar epithelial cells. Previous work has indeed shown that alveolar type II pneumocytes can also become infected with *Mtb* bacteria in vitro and in vivo [13–17]. Furthermore, dendritic cells and neutrophils internalize *Mtb* bacteria and are important key players in the immune response against this pathogen [18–20].

**Figure 1:**
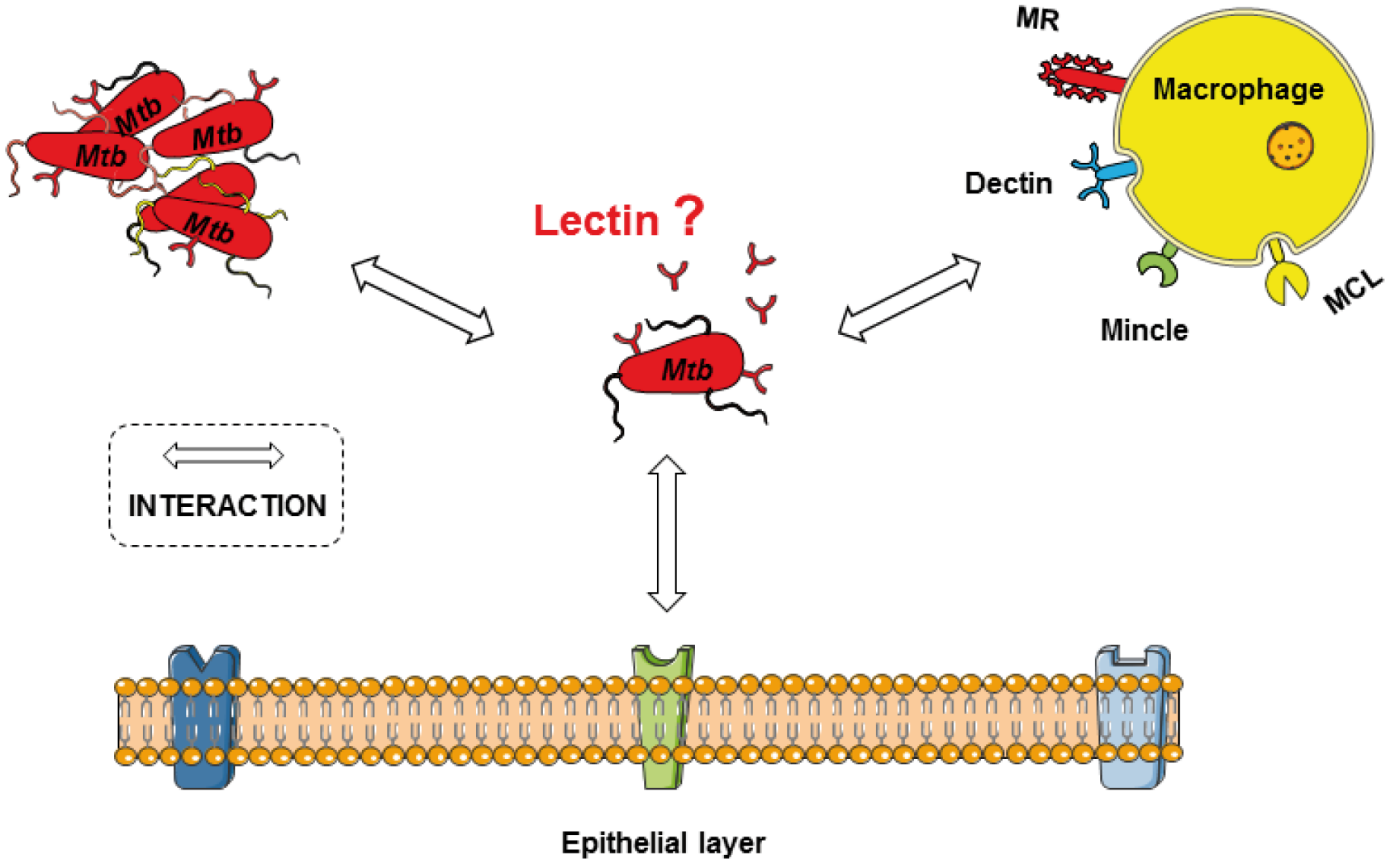
Immune cells (e.g., macrophages) and epithelial cells express lectins on the cell surface (e.g., dendritic cell-specific C-type lectins (Dectin), the macrophage inducible C-type lectin (Mincle), the macrophage C-type lectin (MCL), and the mannose receptor (MR)), which recognize carbohydrates of the *Mtb* cell wall. These proteins contribute to bacterial adhesion and uptake, as well as intracellular survival of the pathogen. The relevance of mycobacterial lectins for host–pathogen interactions has been poorly studied and is the focus of this review.

Bacterial invasion of host cells is a complex process, which is initiated by interactions between host and bacterial cell surface structures. As shown in previous studies, host cells can bind to mycobacterial cell wall carbohydrates via a class of surface-localized or secreted proteins known as lectins, and these interactions strongly contribute to bacterial adhesion and uptake, and are also associated with the capability of *Mtb* to survive, replicate, and persist within macrophages [21–25]. Ubiquitous in both eukaryotes and prokaryotes, lectins comprise a subclass of glycan-binding proteins most commonly associated with intercellular binding, cell–cell recognition, intracellular protein trafficking, and toxin activity [26]. Lectins typically possess high carbohydrate ligand specificity, enabling precise control over protein–target contacts and associated downstream processes. Lectins are often easily identified based on the primary amino acid sequence alone, due to the presence of conserved lectin-associated domains (carbohydrate-recognition domains; CRDs) [27]. Well known lectin examples within the innate immune system include the DC-specific intercellular adhesion molecule 3-grabbing nonintegrin (DC-SIGN) [28–29], the dendritic cell-specific C-type lectins (Dectin-1, Dectin-2) [30–31], the macrophage inducible C-type lectin (Mincle) [32–34], the macrophage C-type lectin (MCL) [35–36], and the mannose receptor (MR) [37–38] (Figure 1). Since the importance of host lectins in *Mtb* infection has already been studied and reviewed in detail [21,39], we focus this review on the rarely studied mycobacterial lectins and their roles in recognizing glycosides on the surfaces of host immune and epithelial cells.

## Review

### Glycosides on the surfaces of mycobacteria and their host cell

**Figure 2:**
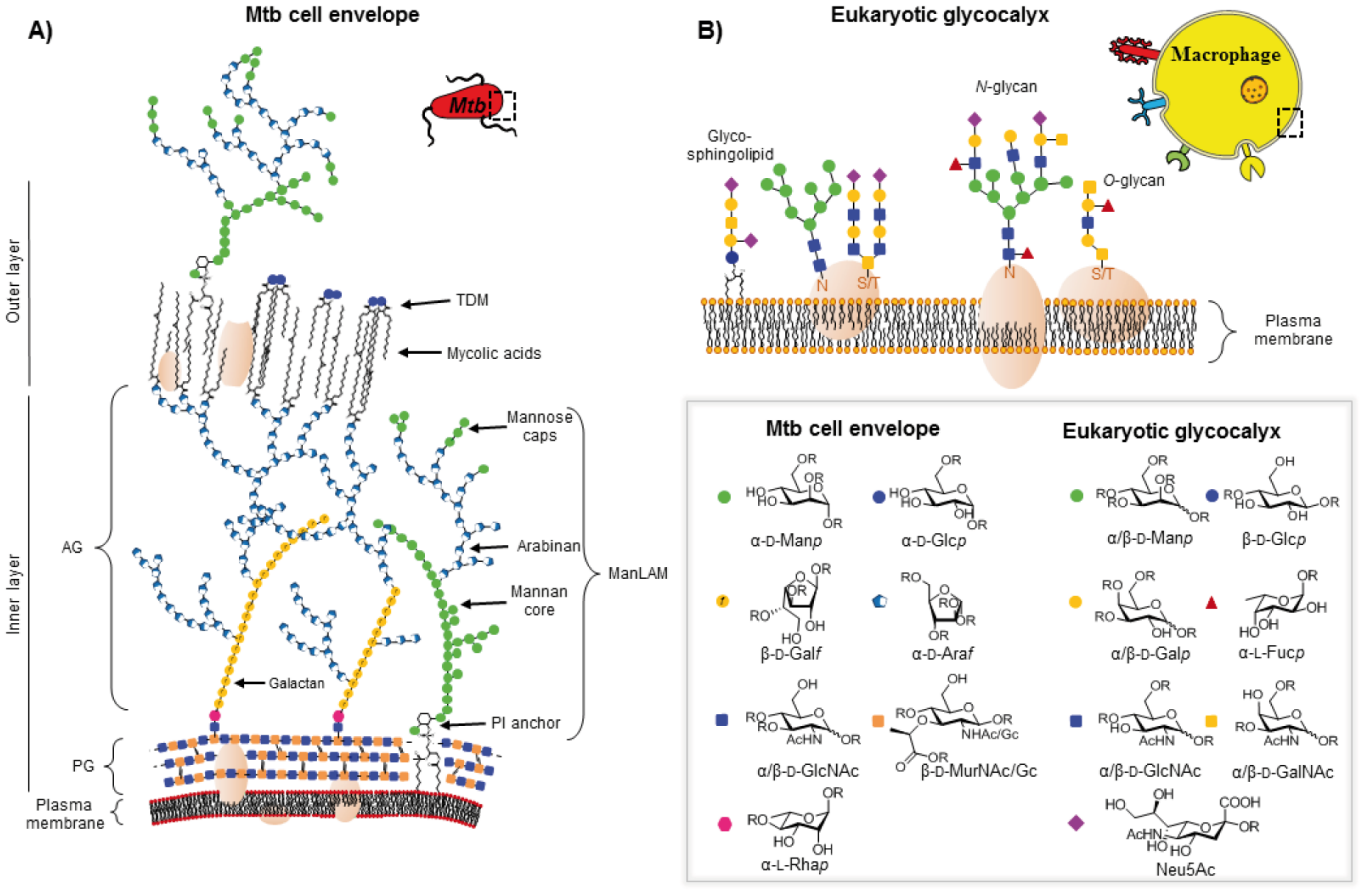
Both mycobacteria and mammalian host cells possess unique subsets of glycosides on their cell surfaces. The main carbohydrates of the *Mtb* cell envelope, a multi-layered structure composed of a mycolyl–arabinogalactan (AG)–peptidoglycan (PG) complex, the lipoglycans lipomannan (LM), and mannosylated lipoarabinomannan (ManLAM), as well as glycolipids, such as trehalose 6,6'-dimycolate (TDM) and trehalose 6-monomycolate (TMM), are α-D-mannopyranosides (α-D-Man*p*), α-D-glucopyranosides (α-D-Glc*p*), α-D-galactofuranosides (α-D-Gal*f*), α-D-arabinofuranosides (α-D-Ara*f*), α-L-rhamnopyranosides (α-L-Rha*p*), *N*-acetyl-α-D-glucosamine (α-D-GlcNAc), *N*-acetyl-β-D-glucosamine (β-D-GlcNAc), and *N*-acetyl- or *N*-glycolyl-β-D-muramic acid (β-D-MurNAc/Gc) residues. The eukaryotic glycocalyx, composed of various glycolipids and glycoproteins, contains D-mannopyranosides (D-Man*p*), D-glucospyranosides (D-Glc*p*), D-galactopyranosides (D-Gal*p*), L-fucopyranosides (L-Fuc*p*), *N*-acetyl-D-glucosamine (D-GlcNAc), *N*-acetyl-D-galactosamine (D-GalNAc), and sialic acid residues, such as *N*-acetyl-D-neuraminic acid (Neu5Ac). While most of the internal glycosides in eukaryotic oligosaccharides are β-linked, terminally localized carbohydrates are often attached via an α-glycosidic bond. (R = H or glycosidic linkage; the figure of the *Mtb* cell wall was originally published in the thesis of K. Kolbe [12] and has been slightly modified for this article).

Eukaryotic cells exhibit a diverse array of glycoconjugates on their cell surfaces, together known as the glycocalyx (Figure 2). Carbohydrate moieties of the eukaryotic glycocalyx mainly exist in the form of oligosaccharide chains covalently linked to proteins or lipids. The most prevalent oligosaccharide modifications of glycocalyx proteins are *N*-glycans (asparagine-linked) and *O*-glycans (serine- or threonine-linked), while glycosphingolipids are the major subclass of glycosylated lipids in the cell membrane of human cells (Figure 2). While many core elements of glycocalyx oligosaccharides are conserved between host proteins and cell types, for example the invariant *N*-acetyl-D-glucosamine or *N*-acetyl-D-galactosamine residues that attach *N*- or *O*-glycans, respectively, to the peptide side chains, the large variety and possible permutations of “capping” residues (for example D-mannopyranosides, D-galactopyranosides, L-fucopyranosides and sialic acids) that comprise the most terminal, and therefore most accessible for lectin recognition, oligosaccharide regions contribute to a vast diversity of possible glycocalyx structures [40–42]. It is known, for example, that the carbohydrate composition of the glycocalyx is a major determinant of cell type, function, and developmental state, and can have serious pathogenic consequences in the event of dysregulation [43].

The glycoside composition of the mycobacterial cell wall differs strongly from the glycocalyx of eukaryotic cells (Figure 2). The bacterial cell membrane is surrounded by a peptidoglycan layer (PG) consisting of multiple, parallel glycan chains of alternating (1→4)-linked subunits of *N*-acetyl-β-D-glucosamine and *N*-acetyl- or *N*-glycolyl-β-D-muramic acid, crosslinked via short conserved oligopeptide stems [44–45]. The PG is covalently attached to the galactan chain of arabinogalactan (AG) by a unique phosphodiester linkage stemming from the 6-OH of a PG muramic acid [46]. AG is the major polysaccharide of the mycobacterial cell envelope and is composed of α-D-arabinosides and β-D-galactosides, both in the relatively uncommon furanose form [47]. The primary hydroxy groups of the terminal arabinofuranoside residues are esterified with mycolic acids forming the basis of the outer lipid layer [48]. The major lipoglycans found in the mycobacterial cell envelope are lipoarabinomannan (LAM) and its precursor lipomannan (LM), both of which consist of a phosphatidyl-*myo*-inositol core structure, glycosylated at the 2-position of *myo*-inositol [49–51]. The oligosaccharide of LM consists exclusively of linear (1→6)-linked and (1→2)-branched α-mannopyranosides [22], while in LAM the mannan structure is elongated by highly (1→2), (1→3) and (1→5)-branched α-D-arabinofuranoside-containing polymers [52]. LAM can further be peripherally modified, also known as ”capping“, the nature of which differs between mycobacterial species. In pathogenic mycobacteria, such as *Mtb*, LAM is capped to various degrees with one to three α-D-mannopyranosides [53], while the fast growing non-pathogenic species *Mycobacterium smegmatis* (*M. smegmatis*) contains inositol phosphate-capped LAM (PILAM) [54]. In addition to lipoglycans, various free, noncovalently associated glycolipids are present in the mycobacterial cell wall, such as the mycolic acid diester trehalose 6,6'-dimycolate (TDM) and its precursor trehalose 6-monomycolate (TMM) [55]. Mycobacteria therefore possess α-D-mannopyranosides, α-D-arabinofuranosides, α-D-glucopyranosides, α-D-galactofuranosides, and their associated oligomeric forms as surface-exposed carbohydrates accessible to extracellular protein recognition. While manno- and glucopyranosides are also present in the eukaryotic glycocalyx, galactofuranosides, arabinofuranosides, and the (1→1)-linked glucose disaccharide trehalose are unique to the mycobacterial cell wall. The occurrence of galactose in the furanose form is restricted to bacteria [56], protozoa [57], and fungi [58], and totally absent in mammals. D-Arabinofuranose can only be found in prokaryotes, for example in Gram-negative bacteria where it is a cytoplasmic intermediate in the biosynthesis of 3-deoxy-D-manno-octulosonic acid (KDO), an essential carbohydrate of the cell wall lipopolysaccharide (LPS) [59]. As a surface-localized carbohydrate, however, D-arabinofuranoside has been exclusively detected in the bacterial suborder of the *Corynebacterineae,* to which the mycobacteria belong [60]. Cell wall-localized D-trehalose is likewise restricted to *Corynebacterineae* [61–62].

In summary, both mycobacteria and mammalian host cells possess unique subsets of surface-exposed carbohydrates, which could function as ligands for putative host- or self-lectins, in processes such as interbacterial aggregation or host–pathogen interactions.

### Bacterial lectins

The existence of bacterially-expressed lectins has been known since the first half of the 20th century. Many of these bacterial lectins were originally detected based on their ability to agglutinate red blood cells. Their primary function, however, is to facilitate adhesion of bacteria to host cells or to contribute to interactions among bacteria, which is crucial for the formation of well-organized superstructures such as biofilms. In contrast to eukaryotic lectins, bacterial lectins commonly occur in the form of filamentous protein appendages projecting from their surface, known as fimbriae and pili [63]. Fimbriae are present in high numbers (100–400) on bacterial surfaces, have a diameter of 5–7 nm and can extend hundreds of nanometers in length. Pili, on the other hand, are thicker, longer, and less abundant. Most bacteria encode multiple lectins, each with different carbohydrate specificities [63]. The most intensely studied bacterial lectins are the mannose-specific FimH of type 1 fimbriae and the galabiose-specific PapG of P fimbriae, expressed by *Enterobactericea*, such as *Escherichia coli* (*E. coli*). While type 1-fimbrial expression of *E. coli* is associated with urinary tract infections, the presence of P fimbriae is connected to colonization of the kidney [64–65]. Inhibition of carbohydrate–lectin interactions by antiadhesive drugs is an emerging anti-infective therapeutic approach, particularly in light of increasing rates of bacterial resistance to traditional antibiotics. α-D-Mannosides containing aromatic aglycons, which act as FimH antagonists, for example, have been successfully used to significantly reduce the severity of *E. coli* infections of the urinary tract in mice [66]. Furthermore, preliminary clinical trials with D-mannose indicate promising effects of this monosaccharide on controlling urinary tract infections in humans, presumably through interference with lectin-associated pathogen–host adhesion [67–68]. Besides facilitation of adhesion, some bacterial lectins are also known to act as toxins. The secreted pertussis toxin, for example, is a lectin and an important virulence factor of *Bordetella pertussis* [69–71], the bacterial pathogen responsible for the respiratory disease pertussis, or whooping cough. While no reports exist to date, inhibiting the adhesion of the pertussis toxin to host–cell surface carbohydrates using carbohydrate ligand mimics might permit reduction of the pathogenicity of the toxin and thereby severity of disease.

### Mycobacterial lectins

The first experimental evidence of the existence of mycobacterial lectins was described in 1989, when Kundu et al. isolated a 12–14 kDa protein with lectin properties (subsequently named “mycotin’) from the culture supernatant of non-pathogenic *M. smegmatis*. This protein was able to agglutinate human A, B and O erythrocytes [72], and the detected hemagglutination could be inhibited by different carbohydrates. The polysaccharide arabinogalactan isolated from *M. smegmatis*, composed of α-D-arabinofuranosides and β-D-galactofuranosides, as well as the monosaccharide D-arabinose, were both found to reverse agglutination, while the α-L-arabinofuranoside-, β-L-arabinopyranoside-, and β-D-galactopyranoside-containing larch wood arabinogalactan and the corresponding L-arabinose monosaccharide were ineffective. Furthermore, the yeast polysaccharide mannan, composed of linear α(1→6)-linked, and α(1→2)- and α(1→3)-branched mannopyranosides, and the glycoside *p*-nitrophenyl α-D-mannopyranoside showed even higher hemagglutination inhibitory potency. These initial experiments let to the assumptions that mycotin is a secreted D-arabinoside- and α-D-mannopyranoside-binding lectin [72]. The relatively high glycoside concentrations (in the mM range) used in this hemagglutination inhibition assays; however, indicate that the tested mono- and polysaccharides are not the optimal or native ligands for this lectin. Other mycobacteria, like *Mtb*, have since been found to contain molecules immunologically related to mycotin on their cell surface [73]. Furthermore, adhesion of *Mtb* to mouse peritoneal macrophages was inhibited using antimycotin antibodies, which led to the assumption that mycotin-like molecules are involved in the interaction of *Mtb* with macrophages and might play a role in *Mtb* infections [73]. However, the 35 kDa cell wall-localized mycotin-like protein identified in *Mtb* in this study was not further characterized and it is still unclear where it is encoded in the bacterial genome. Cell surface-localized mycobacterial lectins and their corresponding ligands have been further investigated using cellular aggregation assays [12,74]. Mycobacteria are known to form large clumps, especially in stationary liquid culture, and it is postulated that lectin–glycan interactions may be at least partially responsible for this aggregation. Anton et al. identified several monosaccharides able to disperse mycobacterial clumps and inhibit bacterial cellular aggregation when added to pure cultures, including D-arabinose (both *M. smegmatis* and *Mtb*), D-xylose, inositol, and D-glucose (*M. smegmatis* only). The impact of D-glucose on *M. smegmatis* aggregation was studied in more detail, where an inhibitory effect of methyl β-D-glucoside, but not methyl α-D-glucoside, was observed [74]. However, the related lectins that mediate self-aggregation have not been isolated or further analyzed to date.

These preliminary findings suggest that *Mtb* has the capacity to express a D-arabinose-specific lectin involved in aggregation processes, and a mycotin-like protein important for adhesion of mycobacteria to macrophages (Table 1).

**Table 1:** Identification and characterization of the lectin mycotin and inhibition studies of bacterial agglutination have provided initial insights into carbohydrate specificity, sub-cellular location and functions of putative mycobacterial lectins.

Mycobacterial species	Carbohydrate specificity	Potential location of the lectin	Potential lectin function

*Mtb*	unknown (maybe mannosides)	cell wall	interaction with macrophages
	D-arabinose	cell surface	agglutination
*M. smegmatis*	D-arabinoside, α-D-mannopyranoside	supernatant	interaction with macrophages
	D-arabinose, D-xylose, inositol, methyl β-D-glucoside	cell surface	agglutination

More recently, microtiter plate-based adherence assays were used to further support the carbohydrate-dependent adhesion characteristics of *Mtb* [12]. The author observed stronger adhesion of *Mtb* H37Rv in wells functionalized with α-D-galactopyranoside **1**, or the Actinobacteria-specific cell wall disaccharide D-trehalose (**2**), compared to β-D-glucopyranoside **3** or α-D-mannopyranoside **4**. In contrast to the results described by V. Anton et al. [74], the bacteria did not adhere to surfaces functionalized with the D-arabinoside derivative **5**. However, in the synthetic structure **5**, arabinose is fixed in the furanose form, while the unmodified D-arabinose, applied by V. Anton et al., is mainly present in the pyranose form. Thus, the results might not be contradictory, but rather suggest that an arabinopyranose-, but not arabinofuranoside-binding lectin might be present in the mycobacterial cell envelope. *M. bovis* BCG bacteria showed divergent and much broader adhesion characteristics with strong binding to α-D-galactopyranoside **1**, trehalose (**2**), β-D-glucopyranoside **3**, α-D-mannopyranoside **4** and D-arabinofuranoside **5**, but not α-D-glucopyranoside **6** (Table 2) [12].

**Table 2:** In the thesis of K. Kolbe various sugar derivatives were immobilized in 96 well microtiter plates via an amino group. Bacterial adhesion was studied using GFP-expressing mycobacterial strains. Stronger fluorescence intensities detected after incubation and washing steps was correlated to a higher amount of bacteria, and therefore stronger adhesion. The experiments verified carbohydrate-dependent adhesion characteristics of *M. bovis* BCG bacteria and *Mtb* H37Rv bacteria. The carbohydrate binding specificity strongly varied between the two investigated mycobacterial species. (+++: very strong adhesion, ++: strong adhesion, +: adhesion, −: no adhesion).

Immobilized carbohydrate derivatives	Adhesion *Mtb*	Adhesion BCG

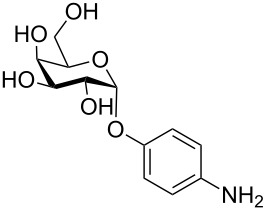 **1**	+++	+
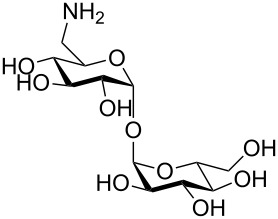 **2**	+++	++
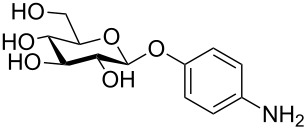 **3**	−	++
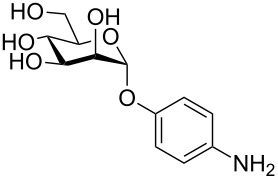 **4**	−	++
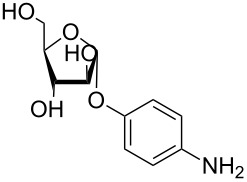 **5**	−	++
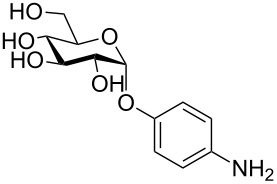 **6**	−	−

**Figure 3:**
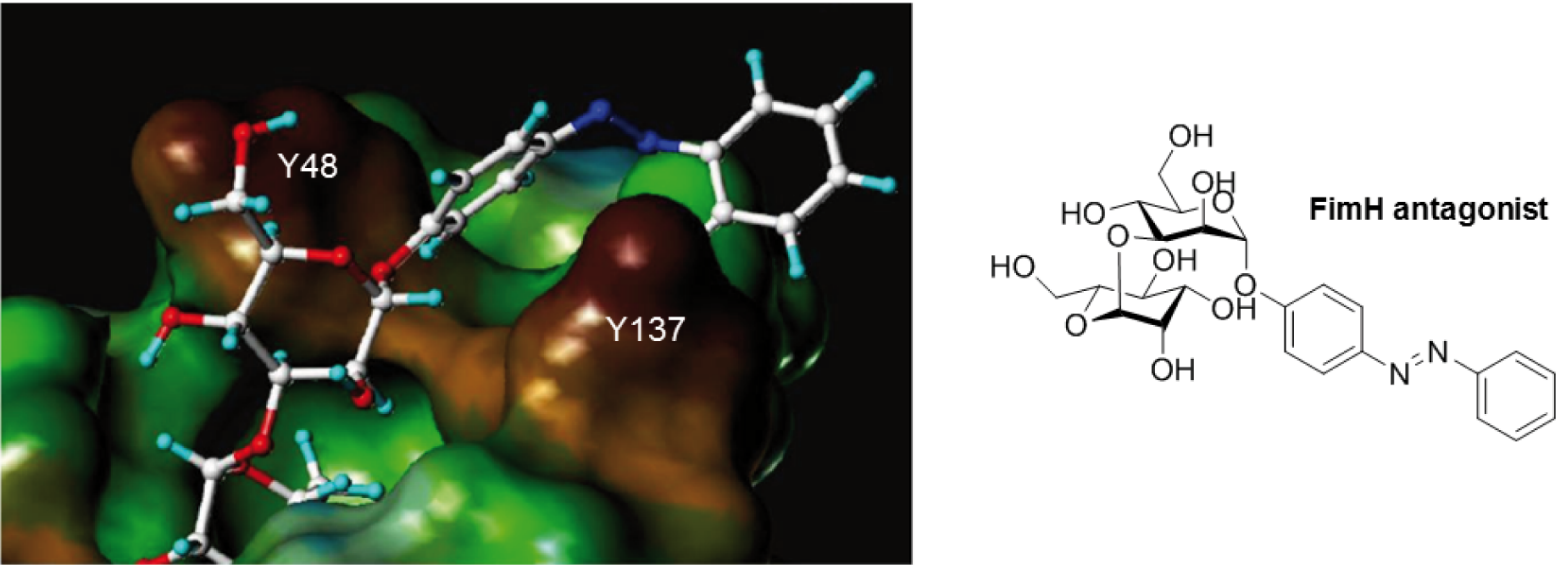
Structure of FimH CRD with a docked azobenzene mannobioside showing the aromatic aglycon and the tyrosine residues, Y48 and Y137, of the protein in close proximity. The figure is a slightly modified version of an image originally published by V. Chandrasekaran et al. [76].

In general, stronger adhesion was detected for carbohydrate derivatives with aromatic aglycon moieties compared to aliphatic aglycons (structures not shown) [12]. These results are similar to previous observations with other lectins. Adhesion and inhibition studies with the fimbrial lectin FimH of *E. coli* bacteria, for example, also revealed higher affinities of glycosides carrying an aromatic aglycon compared to derivatives with aliphatic aglycon portions. This finding can be attributed to π-interactions with tyrosine residues located at the rim of the carbohydrate binding pocket (Figure 3) [64,75].

Importantly, adhesion of *Mtb* was observed to both mycobacterial- and host-specific carbohydrates indicating that cell surface-localized mycobacterial lectins may be involved in mediating both inter-bacterial and bacteria–host interactions. Furthermore, the carbohydrate specificity of *Mtb* adhesion appears to differ significantly from BCG, suggesting that lectins may constitute a contributing factor to the differences in human pathogenicity observed between the two species [12].

**Figure 4:**
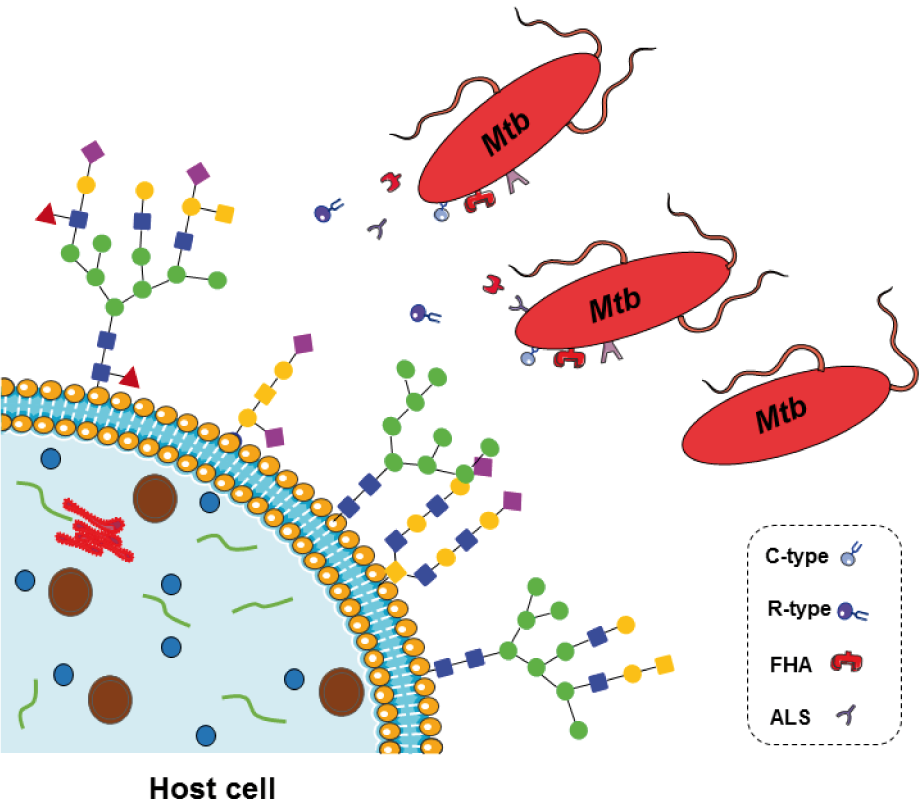
Computer-based genome analysis supports the existence of mycobacterial glycan-binding proteins, which can be associated with known lectin and glycosaminoglycan-binding protein families, including agglutinin-like sequences (ALS), mannose-sensitive hemagglutinin (MSHA), C-type lectins, and R-type lectins, filamentous hemagglutinin (FHA) and heparin-binding hemagglutinin (HBHA). However, hitherto there is only limited information concerning expression, cell localization and function of mycobacterial lectins and glycosaminoglycan-binding proteins.

The presence of mycobacterial lectins was further supported by Abhinav et al. using in silico genome analysis. A bioinformatics homology-based search of lectin-encoding gene regions in 30 fully or partially sequenced mycobacterial genomes identified 94 potential glycan-binding proteins. The number of detected potential lectins, which ranged from one to six per strain, and their phylogenetic association to established lectin families strongly varied depending on the mycobacterial species in question [77]. These results are consistent with the varying carbohydrate-binding characteristics observed between different mycobacterial species, as described above [12,74]. While three potential glycan-binding proteins were identified in the *Mtb* (H37Rv) genome in this study (Table 3) [77], Singh et al., using a different suite of bioinformatic tools, identified eleven, of which nine were annotated as potential lectins [78]. However, most of the proteins encoded by these genes have yet to be biochemically characterized, precluding further functional predictions. Exceptions are the secreted 13 kDa large lectin from *Mtb*, sMTL-13 [79–80], and the heparin-binding hemagglutinin (HBHA) [81–85], which have been previously studied in detail (see below). We subsequently discuss the association of the nine putative *Mtb* lectins identified by Singh et al. and Abhinav et al. with established lectin families [77–78], such as agglutinin-like sequences (ALS), mannose-sensitive hemagglutinin (MSHA), C-type lectins, and R-type lectins. Furthermore, the filamentous hemagglutinin (FHA) and the heparin-binding hemagglutinin (HBHA) as glycosaminoglycan-binding protein families are also discussed (Table 3, Figure 4).

**Table 3:** Eleven *Mtb* genes were predicted based on in silico genome analysis to encode for glycan-binding proteins, as reported by Singh et al. The three genes in bold (Rv2075, Rv1419, Rv0475) were also identified by Abhinav et al., using different bioinformatics methods. Only two of the encoded proteins have been biochemically characterized to date.

Lectin family	Gene ID	*Mtb* protein

agglutinin like sequences (ALS)	Rv2082, Rv1753	–
mannose sensitive hemagglutinin (MSHA)	Rv2813, Rv3659	–
**C-type lectin**	**Rv2075**	–
**R-type lectin**	**Rv1419**	**sMTL-13**
filamentous hemagglutinin (FHA)	Rv0355, Rv1917, Rv3343, Rv3350	–
**heparin-binding hemagglutinin (HBHA)**	**Rv0475**	**HBHA**

#### Agglutinin-like sequences

Based on bioinformatic analysis, the two *Mtb* gene products of Rv1753 and Rv2082 were reported to have 27% and 25% amino acid sequence similarity to the ALS1 gene from *Candida albicans*, which encodes the candida adhesin [78]. This lectin is cell surface-localized and mediates adherence of the fungus to endothelial and epithelial cells [86–87]. Fucose-containing glycans were detected as potential carbohydrate ligands for the ALS1 protein [88]. Intriguingly, Rv1753 is described as essential for in vitro growth of *Mtb*, as detected by transposon mutagenesis studies [89–90]. However, no further biochemical or genetic data are available for either Rv1753 or Rv2082, and an associated ALS-like lectin function is only speculation.

#### Mannose-sensitive hemagglutinin

Two *Mtb* gene products, encoded by Rv2813 and Rv3659, were classified as MSHA-like proteins, with the highest amino acid similarity directed to MshM (41%) and MshE (26%), respectively, of the marine bacterium *Pseudomonas haloplanktis* [78]. These genes encode for proteins involved in assembly of type IV pili (T4P) [91]. Since bacterial lectins are often located at the terminal ends of pili or fimbriae, this homology is of potential interest as it indicates that *Mtb* might express carbohydrate-binding pili on the cell surface (discussed further below).

#### C-Type lectin

**Figure 5:**
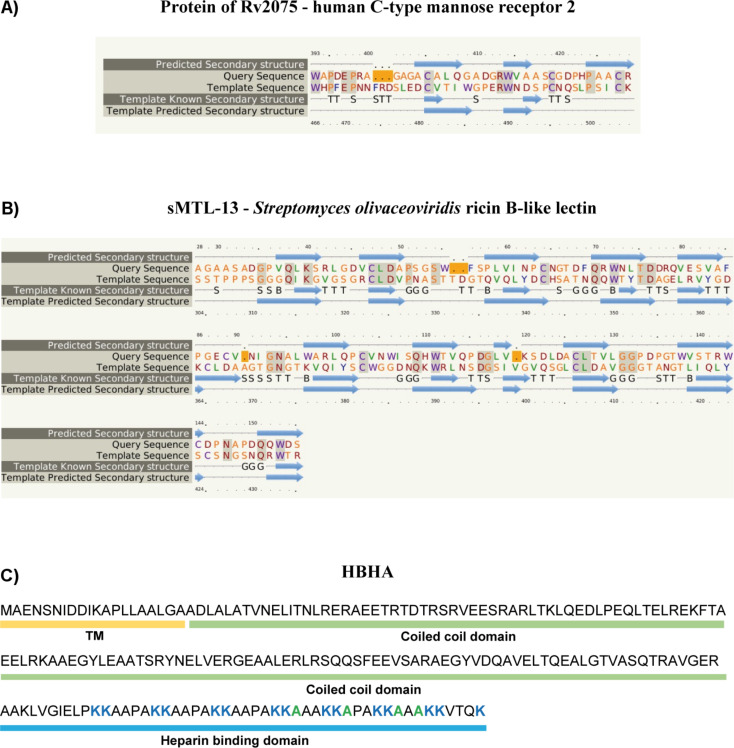
Amino acid sequence and secondary structure alignments of *Mtb* proteins encoded by Rv1419 and Rv2075 to known proteins were determined using Phyre^2^. A) The amino acid sequence (aa393-428) of Rv2075 showed with 97% confidence sequence similarity to the C-type lectin domain of the human C-type mannose receptor 2, the sequence identity was 28%. Identical amino acids are highlighted in grey, amino acids with a small/polar side chain: orange, hydrophobic side chain: green, charged side chain: red, aromatic amino acids and cysteine: violet. β-Sheets of the secondary structure are shown as blue arrows. B) sMTL-13 (aa28-155), encoded by Rv1419, showed with 100% confidence sequence similarity to a ricin B-like lectin of *Streptomyces olivaceoviridis*, the sequence identity was 22%. C) Known domain structure of HBHA (Rv0475): Transmembrane domain (TM), coiled coil domain, and heparin binding domain. Amino acids involved in heparin bind are colored in blue (lysine) and green (alanine).

C-Type lectins are one of the largest and most diverse lectin families, including the *Mtb*-recognizing eukaryotic host immune receptors DC-SIGN, Dectin-1/2, Mincle, MCL, and MR, mentioned before. These lectins bind carbohydrates in a calcium-dependent manner. The ligand specificity is highly diverse, including fucosides, mannosides, glucosides, *N*-acetylglucosamines, galactosides, and *N*-acetylgalactosamines. While some of the C-type lectins are known to be secreted, others are membrane-associated proteins. They often oligomerize into homodimers, homotrimers, and higher-ordered oligomers, which increases their avidity for multivalent ligands. C-Type lectins play key roles in cell–cell interactions, such as host–pathogen interactions, and phagocytosis [92]. The *Mtb* gene product of Rv2075c shows partial amino acid sequence similarity to mannose-specific C-type lectins from *Caenorhabditis elegans*, *Mus musculus*, and *Homo sapiens* (see Figure 5 for partial secondary structure prediction and alignment with the human C-type mannose receptor 2) [77–78], and is predicted to be localized to the outer membrane [93]. While Rv2075c orthologues have been identified in all tested *Mtb* strains (*Mtb* H37Ra, *Mtb* H37Rv, *Mtb* KZN 1435, *Mtb* KZN 4207, *Mtb* CDC1551), no homologous gene was identified in the *Mycobacterium africanum* strain GM041182 [77]. TB in humans is primarily caused by *Mtb*, but can also be a consequence of infection with *Mycobacterium africum,* which is currently limited to West Africa [94]. Thus, the potential C-type lectin of *Mtb* might not be essential for a typical TB infection in humans. However, cell localization and function need to be investigated in further detail.

#### R-Type lectin

R-Type lectins are classified as lectins containing a carbohydrate-recognition domain similar to the CRD in ricin, a toxin of the poisonous plant *Ricinus communis*. R-type lectins have been detected in plants, animals, and bacteria. Plant R-type lectins often contain a separate subunit functioning as a toxin. Furthermore, ricin-type lectin domains have been found in glycosyltransferases as well as in bacterial hydrolases [95]. The *Mtb* gene product of Rv1419 shows 41% amino acid sequence similarity to R-type lectins and encodes the *Mtb* protein sMTL-13 (see Figure 5 for secondary structure prediction and alignment with the ricin B-like lectin from *Streptomyces olivaceoviridis*) [77–78]. This secreted protein was crystallized in 2010 by Patra et al., however, a three-dimensional structure has yet to be resolved [79]. Recently Nogueira et al. detected high titers of IgG antibodies against sMTL-13 in sera from TB patients, a response found to be diminished following successful antituberculosis therapy [80]. The results underline that mycobacterial lectins are expressed in vivo and might be important for *Mtb* infections. Furthermore, anti-sMTL-13 antibodies could serve as a biomarker of disease treatment progression. The exact function of sMTL-13 and its ligand specificity are, however, still unknown. As described before some R-type lectins exhibit toxin activity. Until recently *Mtb* was regarded as a bacteria that does not express toxins [96–98]. In 2014 Danilchanka et al. challenged this paradigm by discovering that the secreted C-terminal domain of the outer membrane channel protein CpnT acts as a toxin [99]. Thus, it might be conceivable that certain *Mtb* lectins could also have toxin function.

#### Filamentous hemagglutinin

One of the most well-characterized FHAs is expressed by *Bordetella pertussis*. The FHA of this pathogen is both surface-exposed and secreted. It functions as an adhesin, where it recognizes and binds to sulfated glycolipids on epithelial host–cell surfaces. The ability of the bacteria to attach to and infect the epithelium of the upper respiratory tract is essential in the pathogenesis of the pertussis organism, underlining the crucial role of this lectin in bacterial physiology [100]. FHA also promotes the formation of biofilms by mediating cell–substrate and interbacterial adhesions [101]. Singh et al. reported that the products of four genes of the *Mtb* strain H37Rv: Rv0355, Rv1917, Rv3343, and Rv3350, show varying levels of amino acid sequence similarities to FHA of *Bordetella pertussis* [78]. However, there is no reported biochemical evidence to date of similar lectin functions for any of these proteins.

#### Heparin-binding hemaglutanin

The HBHA encoded by Rv0475 is the most well-characterized glycan-binding protein in *Mtb*. Using biophysical and biochemical methods the domain structure of HBHA has been determined, and includes a canonical lysine-rich C-terminal heparin binding domain (see Figure 5) [83,102–105], which has been shown to bind sulfated glycoconjugates like heparin, facilitating the adhesion of mycobacteria to epithelial cells, but not to macrophages [81–85]. Furthermore, this transmembrane protein has been associated with mycobacterial aggregation [82,85]. BALB/c mice infected with either wild-type or HBHA-deficient *Mtb* displayed equivalent bacterial lung colonization, but the HBHA-deficient mutant showed reduced dissemination to other regions of the body relative to wild type, suggesting that HBHA plays an important role in extrapulmonary spread [84]. It has also been shown that antibodies directed against HBHA can limit adhesion of mycobacteria to epithelial cells in vitro and in vivo [80,83]. Interestingly, anti-HBHA antibodies have been detected in the sera of TB patients [82]. Thus, a humoral immune response to HBHA might also be connected to a reduced dissemination of *Mtb* from human lungs.

Apart from the potential lectins predicted by in silico genome analysis, a C-type lectin-like carbohydrate binding domain was recently identified to be present in the arabinofuranosyltransferase EmbC (Rv3793), which is involved in the LAM biosynthesis of the *Mtb* cell wall [106]. However, the known function of this protein in arabinogalactan biosynthesis suggests the lectin-like domain to be more associated with catalysis and/or substrate recognition, rather than in a canonical interbacterial or host–pathogen lectin–carbohydrate adhesion role.

As described above, only limited data exists concerning expression, subcellular localization and physiological functions of mycobacterial lectins and glycosaminoglycan-binding proteins to date. However, agglutination-inhibition and adhesion assays, genome analyses, and immunological studies have provided the first indications that glycan-binding proteins might be important mediators of TB infections and *Mtb* pathogenesis. Detection of appendages on the mycobacterial surface, as extensively reviewed by Ramsugit et al. [107], further supports the possible existence of carbohydrate-binding proteins on the cell surface of *Mtb*, since bacterial lectins are often located at the terminal end of fimbriae or pili.

### Mycobacterial pili

**Figure 6:**
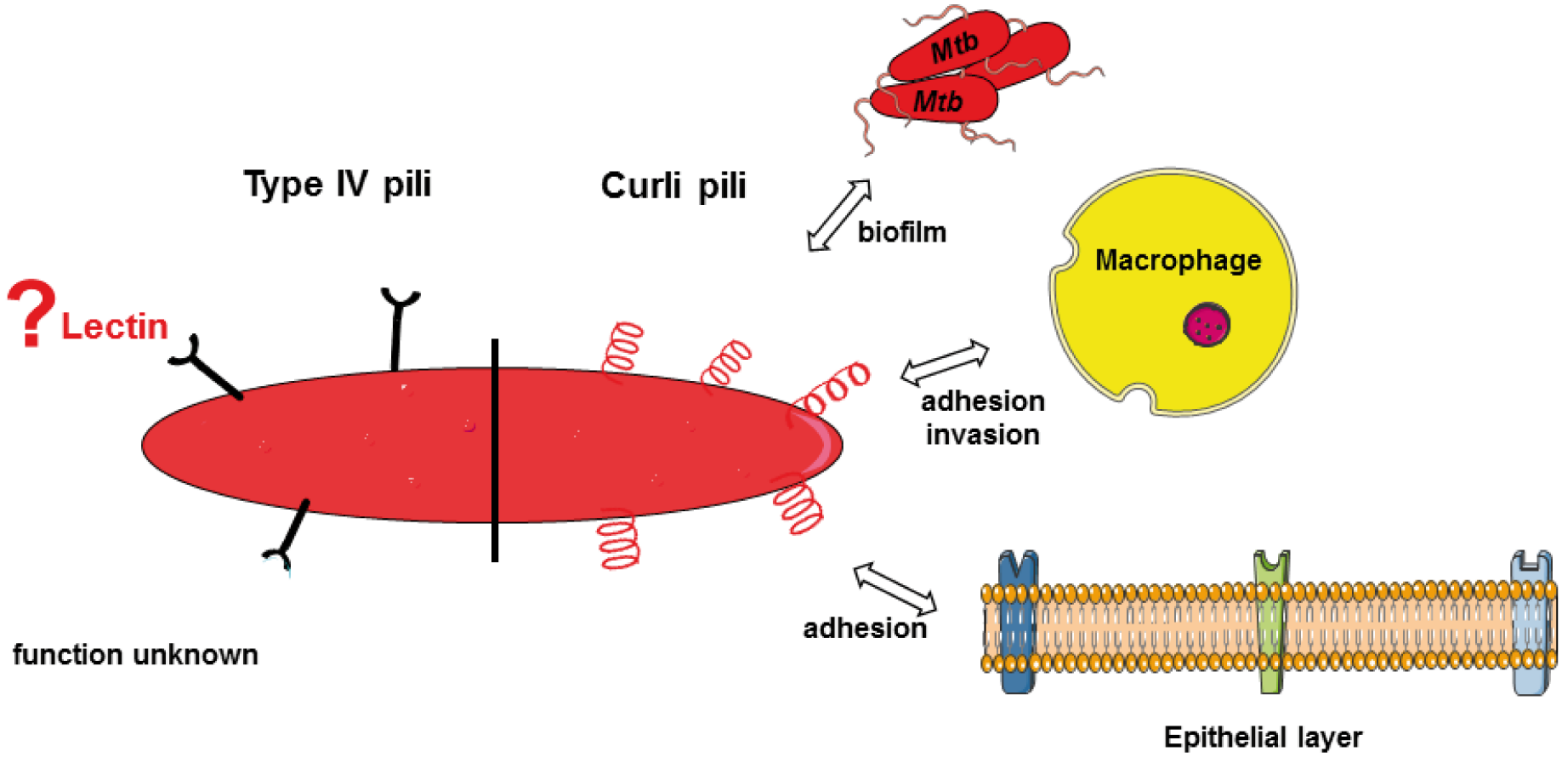
Recently, pili were detected on the cell surface of *Mtb*, which were classified as curli and type IV pili (T4P). While the expression of curli pili is associated with biofilm formation and adhesion to macrophages and epithelial cells, the function of T4P has not yet been examined. Pili often have carbohydrate-binding activity. Whether mycobacterial pili are associated with lectin functions is, however, not known to date.

Mycobacteria have traditionally been regarded as a non-piliated genus; however, recently, studies using transmission electron microscopy (TEM) and atomic force microscopy (AFP) have identified long appendages on the surfaces of *M. smegmatis* and *Mtb*, which could be identified as pili [108–110]. Two different pili types were detected for *Mtb* bacteria (Figure 6). Interestingly, type IV pili are expressed by broth-grown *Mtb*, while curli-like pili are mainly produced by bacilli cultured on solid media [108,111].

#### Curli-like pili

Curli pili are classified as coiled, non-branching proteins with a typical β-sheet-rich structure, 4–6 nm wide and with aggregative properties. These cell surface structures are produced by several members of the *Enterobacteriaceae* family [112]. The *Mtb* curli-like pili (MTP) encoded by Rv3312A, although currently disputed [113], are 2–3 nm in diameter, have a similar ultrastructure to curli pili of *E. coli* or *Salmonella* species [114–115], but lack primary sequence homology and the typical β-sheet secondary structure of curli pili from these latter species [108,116]. The *mtp* gene is present in all strains of the *Mtb* complex (MTBC), but absent in non-tuberculosis mycobacteria and other respiratory pathogens [117]. IgG antibodies have been detected in sera of TB patients indicating that MTP are produced during human TB infections [108,118]. Ramsugit et al. studied the adhesive characteristics of MTP using an MTP-deficient (*mtp*-null mutant) strain of *Mtb* and an MTP-overexpressing complemented strain. It was shown that MTP is associated with *Mtb* aggregation and biofilm formation in vitro [116]. The importance of these interactions in patients, however, has yet to be confirmed, as the association of mycobacterial biofilms with bacterial pathogenesis has not yet been conclusively shown in vivo. Besides mediating interactions among mycobacterial cells, MTP has been shown to play a role in *Mtb* adhesion and invasion of A549 pulmonary epithelial cells and THP-1 macrophages [107,119]. Furthermore, an impact of MTP on histopathology in a mouse model of infection has previously been described [113]. Elsewhere, using purified proteins, Alteri et al. detected laminin as a ligand for MTP [108]. While the exact structure recognized by MTP has yet to be determined, laminin is a glycoprotein and so it is conceivable that MTP binds to mono- or oligosaccharide constituents of this protein.

#### Type IV pili

Type IV pili (T4P) are surface-exposed fibers that mediate many functions in both Gram-positive and Gram-negative bacteria, including motility, adhesion to host cells, biofilm formation, DNA uptake, and protein secretion [120–128]. *Mtb* expresses T4P that appear by electron microscopy as rope-like bundles on the cell surface. Mature T4P are encoded by a seven gene operon, the expression of which is up-regulated during contact with A549 epithelial cells and within macrophages [111,129]. However, their significance in *Mtb* pathogenicity has hitherto not been further investigated. Interestingly, one of the T4P-associated genes is Rv3659, previously identified by in silico genome analysis as coding for a potential mycobacterial lectin (see above) [78]. Although the related protein is most likely involved in pili assembly, it is not inconceivable that T4P have carbohydrate-binding characteristics and are involved in adhesion processes, although this has yet to be proven. The hypothesis is supported by the fact that T4P of other bacteria, for example bundle-forming pili from *E. coli* [130], were shown to have lectin function before.

## Conclusion

Lectins are known to play a fundamental role in mediating and regulating numerous biological processes which are initiated by specific carbohydrate recognition. Much effort has been dedicated to the synthesis of specific lectin ligands in order to study and manipulate lectins. On the other hand, intensive work has been spent on the identification and characterization of lectins. Also in microbe–host cell interactions, specific carbohydrate–lectin interactions are the key to adhesion, microbial colonization as well as to infection. For *Mycobacterium tuberculosis* it is known that the macrophage-associated lectins Dectin and Mincle, for example, specifically interact with *Mtb* cell surface glycans, which in many parts differ significantly from the carbohydrates found in eukaryotic cells. However, in spite of the fact that *Mycobacterium tuberculosis* has been the subject of intense research since its discovery in 1882, many details of carbohydrate–protein interactions in *Mtb* infections are still to be discovered. Significant advances have been made in our fundamental understanding of this bacterium in recent years, but several genes annotated in the *Mtb* genome are still classified as coding for “uncharacterized”, “unknown” or “hypothetical” proteins [131–133] including many of the putative *Mtb* lectins and indeed, *Mtb* lectins have been poorly studied in mycobacteria. This account has thus focused on reviewing the available knowledge on *Mtb* lectins, which are a promising field of research with a diagnostic and therapeutic perspective in the field of tuberculosis. Agglutination-inhibition and adhesion assays, as well as immunological studies have indeed provided the first indications that lectins might play an important and as yet underappreciated role in TB infections, underscoring the necessity of more research into these protein families.

## List of Abbreviations

AG: arabinogalactan; ALS: agglutinin-like sequences; D-Ara*f*: D-arabinofuranoside; CpnT: outer membrane channel protein; CRD: carbohydrate-recognition domain; DC-SIGN: dendritic cell-specific intercellular adhesion molecule 3-grabbing nonintegrin; dectin: dendritic cell-specific C-type lectin; FHA: filamentous hemagglutinin; L-Fuc*p*: L-fucopyranoside; D-Gal*f*: D-galactofuranoside; D-Gal*p*: D-galactopyranoside; D-GlcNAc: *N*-acetyl-D-glucosamine; D-Glc*p*: D-glucopyranoside; HBHA: heparin-binding hemagglutinin; KDO: 3-deoxy-D-manno-octulosonic acid; LAM: lipoarabinomannan; LM: lipomannan; LPS: lipopolysaccharide; D-Man*p*: D-mannopyranoside; MCL: macrophage C-type lectin; Mincle: macrophage inducible C-type lectin; MR: mannose receptor; MSHA: mannose-sensitive hemagglutinin; *Mtb*: *Mycobacterium tuberculosis*; MTP: *Mtb* curli-like pili; *M. smegmatis*: *Mycobacterium smegmatis*; D-MurNAc/Gc: *N*-acetyl- or *N*-glycolyl-D-muramic acid; Neu5Ac: *N*-acetyl-D-neuraminic acid; PG: peptidoglycan; L-Rha*p*: L-rhamnopyranosides; sMTL: secreted 13 kDa large lectin from *Mtb*; TB: tuberculosis; TDM: trehalose 6,6'-dimycolate; TMM: trehalose 6-monomycolate; T4P: type IV pili.
